# Intratumoral injection of IFN-alpha dendritic cells after dacarbazine activates anti-tumor immunity: results from a phase I trial in advanced melanoma

**DOI:** 10.1186/s12967-015-0473-5

**Published:** 2015-05-02

**Authors:** Carmela Rozera, Giancarlo Antonini Cappellini, Giuseppina D’Agostino, Laura Santodonato, Luciano Castiello, Francesca Urbani, Iole Macchia, Eleonora Aricò, Ida Casorelli, Paola Sestili, Enrica Montefiore, Domenica Monque, Davide Carlei, Mariarosaria Napolitano, Paola Rizza, Federica Moschella, Carla Buccione, Roberto Belli, Enrico Proietti, Antonio Pavan, Paolo Marchetti, Filippo Belardelli, Imerio Capone

**Affiliations:** Department of Hematology, Oncology and Molecular Medicine, Istituto Superiore di Sanità, viale Regina Elena 299, Rome, 00161 Italy; IV Dermatology Oncology Unit, Istituto Dermopatico dell’Immacolata, Istituto di Ricovero e Cura a Carattere Scientifico (IDI-IRCCS), via Monti Creta 104, Rome, 00167 Italy; Immunohematology and Transfusion Medicine Unit, Sapienza University of Rome, Sant’Andrea Hospital, via di Grottarossa 1035, Rome, 00189 Italy; National AIDS Center, Istituto Superiore di Sanità, viale Regina Elena 299, Rome, 00161 Italy; Department of Oncology, Sapienza University of Rome, Sant’Andrea Hospital, via di Grottarossa 1035, Rome, 00189 Italy

**Keywords:** Advanced melanoma, Dendritic cells, Dacarbazine, Chemo-immunotherapy

## Abstract

**Background:**

Advanced melanoma patients have an extremely poor long term prognosis and are in strong need of new therapies. The recently developed targeted therapies have resulted in a marked antitumor effect, but most responses are partial and some degree of toxicity remain the major concerns.

Dendritic cells play a key role in the activation of the immune system and have been typically used as *ex vivo* antigen-loaded cell drugs for cancer immunotherapy.

Another approach consists in intratumoral injection of unloaded DCs that can exploit the uptake of a wider array of tumor-specific and individual unique antigens. However, intratumoral immunization requires DCs endowed at the same time with properties typically belonging to both immature and mature DCs (i.e. antigen uptake and T cell priming). DCs generated in presence of interferon-alpha (IFN-DCs), due to their features of partially mature DCs, capable of efficiently up-taking, processing and cross-presenting antigens to T cells, could successfully carry out this task. Combining intratumoral immunization with tumor-destructing therapies can induce antigen release *in situ*, facilitating the injected DCs in triggering an antitumor immune response.

**Methods:**

We tested in a phase I clinical study in advanced melanoma a chemo-immunotherapy approach based on unloaded IFN-DCs injected intratumorally one day after administration of dacarbazine. Primary endpoint of the study was treatment safety and tolerability. Secondary endpoints were immune and clinical responses of patients.

**Results:**

Six patients were enrolled, and only three completed the treatment. The chemo-immunotherapy was well tolerated with no major side effects. Three patients showed temporary disease stabilization and two of them showed induction of T cells specific for tyrosinase, NY-ESO-1 and gp100. Of interest, one patient showing a remarkable long-term disease stabilization kept showing presence of tyrosinase specific T cells in PBMC and high infiltration of memory T cells in the tumor lesion at 21 months.

**Conclusion:**

We tested a chemo-immunotherapeutic approach based on IFN-DCs injected intratumorally one day after DTIC in advanced melanoma. The treatment was well tolerated, and clinical and immunological responses, including development of vitiligo, were observed, therefore warranting additional clinical studies aimed at evaluating efficacy of this approach.

**Trial registration:**

Trial Registration Number not publicly available due to EudraCT regulations: https://www.clinicaltrialsregister.eu/doc/EU_CTR_FAQ.pdf

## Introduction

The outcome of patients with advanced melanoma remains bleak. Patients with stage IV disease have poor long term prognosis, with 50-70% surviving less than one year and with 5-year survival rates of approximately 10% [1]. Before 2011, in the pre anti-BRAF/anti-CTLA4 and, more recently, anti-PD1 era, the reference chemotherapeutic agent for the treatment of metastatic melanoma was Dacarbazine (DTIC) with an activity in term of overall response rates (ORR) ranging from 5% to 12% [2]. However, complete responses were observed in less than 5% of patients, the most of them being not durable [3]. Analogous conclusion can be drawn with cytokine-based therapies (i.e. IFN-α or IL-2) or combined chemo-biotherapies which, although improved the response rate in the range of 15-20%, did not result in increased overall survival [3,4]. Recently, targeted therapies based on the use of selective inhibitors of MAPK pathway enzymes in patients showing activating mutations have resulted in a marked antitumor effect, but most responses are partial and disease progression is typically seen at a median of 5–7 months [5]. Notably, immunotherapy has recently re-emerged has a powerful approach for the treatment of advanced melanoma as a result of the registration of a novel type of monoclonal antibodies (ipilimumab, anti-CTLA4; pembrolizumab, anti-PD1) targeting the immune checkpoints [6]. However, while such therapy can result in a significant improvement of survival rate in the range of 22% at 3 years, [7,8] the lack of response in the majority of patients and some degree of toxicity remain the major concerns.

DCs represent the professional cells involved in the induction and regulation of immune response and, during the past years, DC-based immunotherapy has emerged as a promising approach for treating cancer [9]. However, the results of several clinical studies carried out in cancer patients, and in particular in melanoma patients, have also highlighted many critical aspects (type of antigen, DC generation method and maturation status, use of adjuvant, route of vaccine administration, vaccine- induced immune response, combination with conventional therapies) whose definition is of utmost importance in order to enhance the efficacy of DC-based treatment [10]. Notably, DC-based trials have been generally performed by using monocyte-derived DCs generated *in vitro* in the present of IL-4 and GM-CSF and further treated with different types of stimulation factors [11].

IFN-α has been proven to induce the rapid differentiation of GM-CSF-treated human monocytes into partially mature DCs (IFN-DCs) [12-14] endowed with potent functional activities [12,15,16]. IFN-DCs produce mostly T-helper-1 (Th-1) cytokines and chemokines, express toll-like receptors (TLRs) 1 to 8, show migratory response to chemokines, and are capable of stimulating Th-1 polarized immune responses after injection into severe combined immunodeficient mice reconstituted with human peripheral blood leukocytes [14,15]. Notably, IFN-DCs exert a direct cytotoxic effect on tumor cells, [12] are capable to take up, through the scavenger receptor Lectin-like oxidized-LDL receptor-1 (LOX-1), apoptotic cells [17] and cross-present their antigens to CD8^+^ T cells, thus leading to an efficient cross-priming of these cells [18-20]. In addition, IFN-DCs are capable of expanding both Th-1 and Th-17 responses as a result of the production of cytokines such as IL-23 and IL-12 [21]. Remarkably, IFN-DCs do not require TLR triggering to induce antigen specific cytotoxic T lymphocytes and to stimulate allogeneic CD4^+^ T cells [22]. All these features make IFN-DCs highly promising new candidates for the development of more effective DC-based strategies of cancer immunotherapy [23,24].

For typical active immunotherapy strategies, DCs are generated *ex vivo* from monocytes, pulsed with tumor antigens, and then injected into patients. Another approach consists in intratumoral injection of unloaded DCs, which has been tested in experimental models [25,26] as well as in humans [27,28]. This approach can exploit the uptake by the DCs of a wide array of tumor-derived antigens, including tumor-specific and individual unique antigens, and their DC-mediated presentation to the immune system, possibly resulting in the redirecting of tumor-specific responses back to the tumor site. However, the intratumoral immunization requires DCs endowed at the same time with properties typically belonging to both immature and mature DCs (i.e. antigen uptake and T cell priming), unless immature DCs more competent to capture antigens are used and co-delivered with a maturation stimuli necessary for effective T cell activation. IFN-DCs, due to their features of partially mature DCs, capable of efficiently up-taking, processing and cross-presenting antigens to T cells, can successfully carry out this task [24].

Combining intratumoral immunization with tumor-destructing therapies can induce antigen release *in situ*, facilitating the injected DCs in triggering an antigen-driven immune response [29]. Indeed, intratumoral DC-based immunotherapy has been used in combination with systemically administered antitumor drugs [30-34] as well as locally targeted therapies such as radiation therapy [35-40] on the basis of recent evidence indicating that both cytotoxic drugs and radiation induce a form of tumor cell death that is immunologically active, therefore facilitating an adaptive immune response [41]. Moreover, cytotoxic drugs can promote antitumor immune responses by altering the tumor-induced tolerogenic mechanisms occurring within the tumor microenvironment [42].

Herein, we investigated in a phase I clinical trial a chemo-immunotherapy combination approach based on IFN-DCs injected into metastatic tumor lesions of patients with advanced melanoma pre-treated one day before with DTIC, with the aim of triggering a strong and long-lasting immune response against melanoma antigens released in situ, as a consequence of the chemotherapy-induced cell death, and possibly taken up by the locally injected IFN-DCs.

## Materials and methods

### Study design, patient selection and treatment

This was a phase I clinical trial, approved by the Ethical Committees of Istituto Dermopatico dell’Immacolata (IDI) of Rome and conducted according to Good Clinical Practice and Declaration of Helsinki principles. Subjects were included in the study only after having given their written, informed consent and having carried out all the procedures for complying inclusion/exclusion criteria. Main criteria for eligibility included histologically confirmed malignant stage IV melanoma not suitable for locoregional therapies with unresectable lesions and suitable for chemotherapy; ECOG performance status of 0–1, adequate blood cell counts and kidney-liver function, written informed consent. Patients were excluded if they had a concomitant or previous history of malignant diseases, severe cardiovascular disease, clinically active infections and/or significant autoimmune diseases.

Once enrolled, each patient underwent leukapheresis to provide PBMCs from which autologous IFN-DCs were prepared. Primary end-points were safety and tolerability of the treatment as well as the antitumor T-cell responses. Secondary endpoint was the determination of response rates and survival. Safety and tolerability of the treatment were assessed by determining frequency, type, and intensity of adverse events as well as the patient compliance. T cell responses were assessed by determining magnitude and quality of peripheral tumor-specific CD8^+^ T cell response. The clinical response was evaluated by determining time to progression (TTP) and objective response (OR), according to RECIST/modified WHO criteria. Before, during, and after treatment, blood samples and tumor biopsies were drawn for determining safety as well as potential efficacy parameters. During treatment, patient clinical status was monitored at each visit and any disease progression was checked by appropriate diagnostic investigations at specific time points. Patients showing disease progression during the treatment period were withdrawn from the study and considered not assessable for analysis.

### GMP preparation of IFN-DCs

Leukapheresis was performed by a Fresenius Com-Tech blood cell separator (Fresenius Kabi, Friedberg, Germany) using the White Blood Cell Set (P1YA) for the collection of mononuclear cell (MNC) products. Monocytes enrichment from aphaeresis was performed according to Elutra® Cell Separation System Monocytes Enrichment Protocol. The monocyte enriched fraction was analysed for cell viability and cell counts and purity were assessed by flow cytometry using CD14 mAb associated with the pan leukocyte CD45 mAb (all from BD Biosciences, San Jose, CA). When the purity of monocytes was less than 60%, an additional step of separation, by centrifugation on an isosmotic medium with a density of 1.077 g/ml as a Lymphoprep™ (Axis-Shield, Oslo, Norway), was performed. The enriched monocytes were cultured for three days in bags (Afc/American Fluoroseal Corporation, Gaithersburg, MD) at the concentration of 2×10^6^ cells/ml in a culture medium (CellGenix GmbH, Freiburg, Germany) containing GM-CSF (600 IU/ml) (Leukine sargramostim, Bayer Healthcare Pharmaceuticals, Seattle, WA) and IFN-α2b (10,000 IU/ml) (Merck Sharp & Dohme Limited, Hoddesdon, UK).

IFN-DCs were then harvested, counted and re-suspended in freezing medium, prepared by mixing 9 volumes of 5% Human Serum Albumin (HSA) (Baxter S.p.A., Rome, Italy) + 1 volume of DMSO (WAK-Chemie Medical GmbH, Steinbach, Germany), at the final concentration of 1-2×10^7^ cells/ml. Aliquots of 0.5 ml cell suspension were transferred to 2 ml cryo-vials, that were deep-frozen under decreasing controlled temperature conditions and stored in liquid nitrogen vapor phase.

### Characterization of IFN-DCs

Cell count, viability and recovery were evaluated by using trypan blue staining, counted into at least two large different squares of the Neubauer chamber. The viability was calculated as viable Cell Density × 100/total Cell Density. The recovery was evaluated as a ratio between the number of thawed viable IFN-DCs over the number of frozen viable IFN-DCs. Sterility was determined by Direct Inoculation technique and endotoxin status was evaluated by the LAL test. Immunophenotype of IFN-DCs was analyzed by flow cytometry using a panel of antibodies specific for HLA-ABC, HLA-DR, CD45, CD11c, CD1a, CD86, CD83, CD80, CD40 and CD14 (all from BD Biosciences, San Jose, CA). The capability of IFN-DCs to phagocytize antigens was verified by flow cytometry using OVA conjugated with fluorescein (OVA-FITC) (Molecular Probes, Inc., Eugene, OR). Flow cytometry was carried out with a FACSCanto flow cytometer and the data were analyzed using the FACSDiva software (BD Bioscience, San Jose, CA). IFN-DCs release criteria were: cell viability >70%, cell recovery >50%, antigen uptake >30%, CD80+ >80%, CD86+ >50%, CD83+ >10%, HLA-DR+ > 80%, HLA-ABC+ >80%, CD14+ <65%, CD14 MFI <1000.

### Gene expression profiling

Total RNA was isolated from at least 5 million cells using RNeasy kit (Qiagen, Valencia, CA, USA) for both monocytes and IFN-DCs before and after cryopreservation/thawing. After passing quality control assessment of integrity of purity analyzed with ND-1000 Spectrophotometer (NanoDrop Technologies, Wilmington, DE, USA) and Agilent 2100 Bioanalyzer (Agilent Technologies, Waldbronn, Germany), RNA was amplified and labeled using Agilent Low Input Quick-Amp Labeling Kit, one color (Agilent Technologies) in the presence of cyanine 3-CTP according to manufacturer’s instructions and hybridized on Agilent Chip (SurePrint G3 Human GE 8×60K Microarray) at 65°C for 17 hours. At the end of the hybridization, chips were washed following manufacturer’s instructions and scanned on SureScan Microarray Scanner (Agilent) and images analyzed using Agilent Feature Extraction Software. Data were then analyzed with BRB Array Tools (http://linus.nci.nih.gov/BRB-ArrayTools.html ). The processed data set was subjected to filtration based on signal intensity, spot quality and presence across the data set.

Unsupervised hierarchical clustering was performed using centered correlation and average linkage on average corrected values. Paired statistical analysis of genes differentially expressed between IFN-DCs and monocytes was performed on BRB using Random Variance Model to increase accuracy of variance estimation across the data set. False Discovery Rate was calculated as the proportion of expected false positive observations on total significant observations as described by Sorić [43]. Gene Ontology (GO) analysis was performed on genes statistically differentially expressed (p-value < 0.001) showing a ratio on expression level IFN-DCs/monocytes greater than 3 using the Database for Annotation, Visualization and Integrated Discovery (DAVID, http://david.abcc.ncifcrf.gov ) [44].

### Detection of antigen-responsive T cells

Before, during and after completion of the therapy blood samples from treated patients were collected in lithium-heparin BD Vacutainer® vials (Becton, Dickinson and Company, Franklin Lakes, NJ). Peripheral blood was diluted 1:2 in PBS and stratified on LymphoprepTM (Axis-Shield PoC AS, Oslo, Norway for Fresenius KabiNorge AS). PBMCs obtained after density gradient centrifugation were frozen at −80°C and stored in liquid nitrogen until testing.

T cell responses against melanoma associated antigens (Melan-A/MART-1, NY-ESO-1, MAGE-A1, MAGE-A3, gp100, Tyrosinase, Survivin) were assessed by proliferation assays by using protein-spanning of overlapping peptides (PepMix™, JPT Peptide Technologies GmbH, Berlin, Germany). Two PepMix™ Peptide Pools (HCMVA (pp65) and actin), SEB (Sigma-Aldrich, St. Louis, MO), and PPD (Staten Serum Institute, Copenhagen, DK, Danish public Institution) were used as controls. Briefly, 2 × 10^5^ PBMCs were cultured in triplicate in CellGro® medium (CellGenix GmbH,Freiburg, Germany) supplemented with 1% penicillin-streptomycin in 96-well flat-bottom trays (Corning Costar®, Tewksbury, MA) in the presence of a single antigen-specific peptide pool (1 ug/ml). Cell cultures were conducted for 7 days and [methyl-3H]thymidine (specific activity, 5 Ci/mmol; Hartmann Analytic GmbH, Braunschweig, Germany) was added at a final concentration of 0.5 μCi/well 18 h before harvesting. DNA synthesis was evaluated by counting [methyl-3H]thymidine incorporation with a TriluxMicrobeta counter (Perkin-Elmer, Waltham, MA). Stimulation index (SI) was calculated as the ratio between counts per min (cpm) of the antigen-stimulated lymphocyte culture and mean cpm of cultures stimulated with actin or two non-activating peptide pools or unstimulated. Proliferative response was considered positive when SI was ≥ 2. Samples from the same patient were analyzed simultaneously.

### Tumor specific T cell lines and intracellular staining

Tumor specific T cell lines were generated by *in vitro* stimulation of cryopreserved PBMCs isolated at baseline and post-therapy time points. Briefly, cells were thawed, counted and cultured for 12 days in the presence of IL-2 and IL-7 (added every 2/3 days during the culture) and with the above mentioned peptides pools (1 μg/ml). Half dose of each peptide pool was added at day 7 of culture. IL-2 was removed from the medium 3 days before test (i.e., day 9). On day 12 cells were harvested and assessed for CD69 expression (activation) and for the production of IFN-γ by intracellular cytokine staining (ICS) as previously described [45]. Before ICS, each sample of *in vitro* expanded cells was labelled with HLA-A2*0201 peptide phycoerythrin (PE) multimer complexes specific for Melan-A/MART-1, NY-ESO-1, Tyrosinase, gp100 (see above), then washed and cultured at a 4:1 ratio with autologous IFN-DCs pulsed or not with the peptide pools (1 ug/ml) for 1 hour at 37°C. After the addition of brefeldin A (Golgi Plug) and monensin (Golgi stop) (Becton Dickinson, San Jose, CA, USA), cells were incubated for additional 5 hours. Following the 6 hour stimulation time, final 2 mM EDTA was added to each well and incubated for 15 min. Cells were then incubated for 30 min at 4°C with a 50 μl antibody cocktail containing the surface antigens anti-CD3 APC H7, anti-CD4 Alexa Fluor 488, anti-CD8 Alexa Fluor 647, anti-CD69 PERCP CY5.5 (BD Biosciences). After surface staining cells were fixed/permeabilized with the BD intrasure kit reagents (BD Biosciences) according to the manufacturer’s intructions in order to enable intracellular staining with anti-interferon-gamma (IFN-γ) PE CY7 (BD Biosciences). Cells were then washed, resuspended in paraformaldehyde 2%, acquired on a FACSCanto flow cytometer instrument (BD Biosciences) and analyzed by FACSDiva and/or FlowJo software, version 10 (Tree Star, Ashland). Negative control included cells cocultured with unpulsed IFN-DCs, while *Staphylococcus* enterotoxin B (SEB; Sigma-Aldrich, Munich, Germany, used at 2 μg/mL) was used as positive control.

### Cytokine assays

Supernatants of lymphoproliferation cultures were collected at day 6, just before adding [methyl-3H]thymidine, immediately frozen at −20°C and, once thawed, assayed in the same plate by Bio-Plex® Multiplex System, Bio-Rad Laboratories, Inc. (Hercules, CA). Assay was performed by a custom kit for simultaneous detection of: IL-1β, IL-2, IL-4, IL-6, IL-8, IL-10, IL-12, IL-17A, IFN-γ, TNF-α and GM-CSF and by a kit for IFN-α.

### Immunofluorescence and confocal microscopy

Immunofluorescent labeling was performed on Formalin Fixed Paraffin Embedded (IF-FFPE) tissue sections. Slides (5 μm thick) were deparaffinized, hydrated through graded alcohols and subjected to a Heat-induced Epitope Retrieval step by citrate buffer pH 6,0 (Novus Biologicals) for 3×3 min in microwave. Sections were washed with PBS-T and blocked in PBS-BSA 3% for 30 min at 37°C. Primary antibody (rabbit anti-human CD45, Abcam; mouse anti-human CD45RO, Dako; rabbit anti-human CD68, Abbiotec) was added in PBS-BSA 3% and incubated at 37°C for 30 min. After washing, sections were incubated for 30 min at 37°C with secondary antibodies (AlexaFluor 594 goat anti-rabbit Molecular Probes; AlexaFluor 488 goat anti-mouse; Invitrogen) plus DAPI. Sections were mounted in Vectashield antifade medium (Vector Laboratories) and fluorescence images were taken by Leica confocal microscope.

## Results

### Trial design and patient characteristics

**Figure 1:**
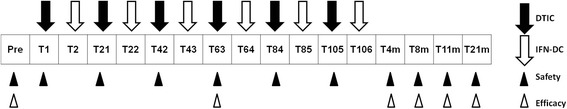
Schedule of treatment regimen and blood samples for safety and efficacy endpoints evaluation. T: time point expressed as days or months (m). Pre: pre-treatment time point.

The study was designed to assess toxicity and immunogenicity of a DC-based immunotherapy associated with standard chemotherapy. From July 2011 to March 2013, ten patients with confirmed diagnosis of metastatic melanoma [46] were screened for their compliance to the study inclusion/exclusion criteria and six of them were enrolled (Table 1). With the exception of one patient presenting unresectable stage IIIC melanoma, all the others were stage IV. Age of patients ranged between 38 and 73 and three of the six were females. Treatment regimen consisted of six administrations of DTIC (1000 mg/m2 i.v. every 3 weeks) each followed one day later by intratumoral injection of ten million unloaded autologous IFN-DCs according to the schedule illustrated in Figure 1. Three out of the six patients completed the treatment (DTIC + injections of DCs) and were assessed for immune and clinical responses, while three patients were excluded because of a rapid disease progression (two patients received two injections of DCs and one progressed before the first vaccination). Four out of five enrolled patients received previous treatments, including immune checkpoint and/or BRAF inhibitors. Patients’ characteristics are listed in Table 1.

**Table 1:** **Patient characteristics and clinical outcomes**

**Patient**	**Sex**	**Age**	**Stage**	**Site of metastasis**	**Previous therapies (drug)**	**Enrolled**	**Vaccine treatment**	**OR**	**TTP (months)**	**Overall survival (months)**
1	F	71	IIIC	Skin recurrence	None	Yes	Complete	SD	7	34
2	F	47	IV	Lung, lymphonodal	2 (DTIC, Vemurafenib)	Yes	2 injections	PD	2	2
3	M	73	IV	Skin lymphnodal, liver	3 (DTIC, Vemurafenib, Ipilimumab)	Yes	Complete	SD	26	31
4	M	38	IV	Lymphonodal, lung	1(Vemurafenib)	Yes	2 injections	PD	2	5
5	M	66	IV	Lung, lymphonodal	3 (DTIC-IL2, BOLD, Ipilimumab)	Yes	Complete	SD	5	9
6	F	68	IV	Skin, lung, lymphonodal	2 (DTIC, Ipilimumab	Yes	No injection	NA	NA	NA
7	F	63	IV	Adrenal, lymphonodal	none	No				
8	M	69	IV	Brain, lymphonodal, lung, bone	3(Fotemustine, Ipilimumab, DTIC)	No				
9	F	51	IV	Brain, lung, lymphonodal, spleen	5 (DTIC, Fotemustine, Paclitaxel, Ipilimumab, Dabrafenib	No				
10	M	66	IV	Lung, lymphonodal, spleen	4 (DTIC+ Bevacizumab, Fotemustine, CDDP + Paclitaxel, Ipilimumab)	No

### Phenotypic and molecular features of IFN-DCs used in the clinical trial

**Figure 2:**
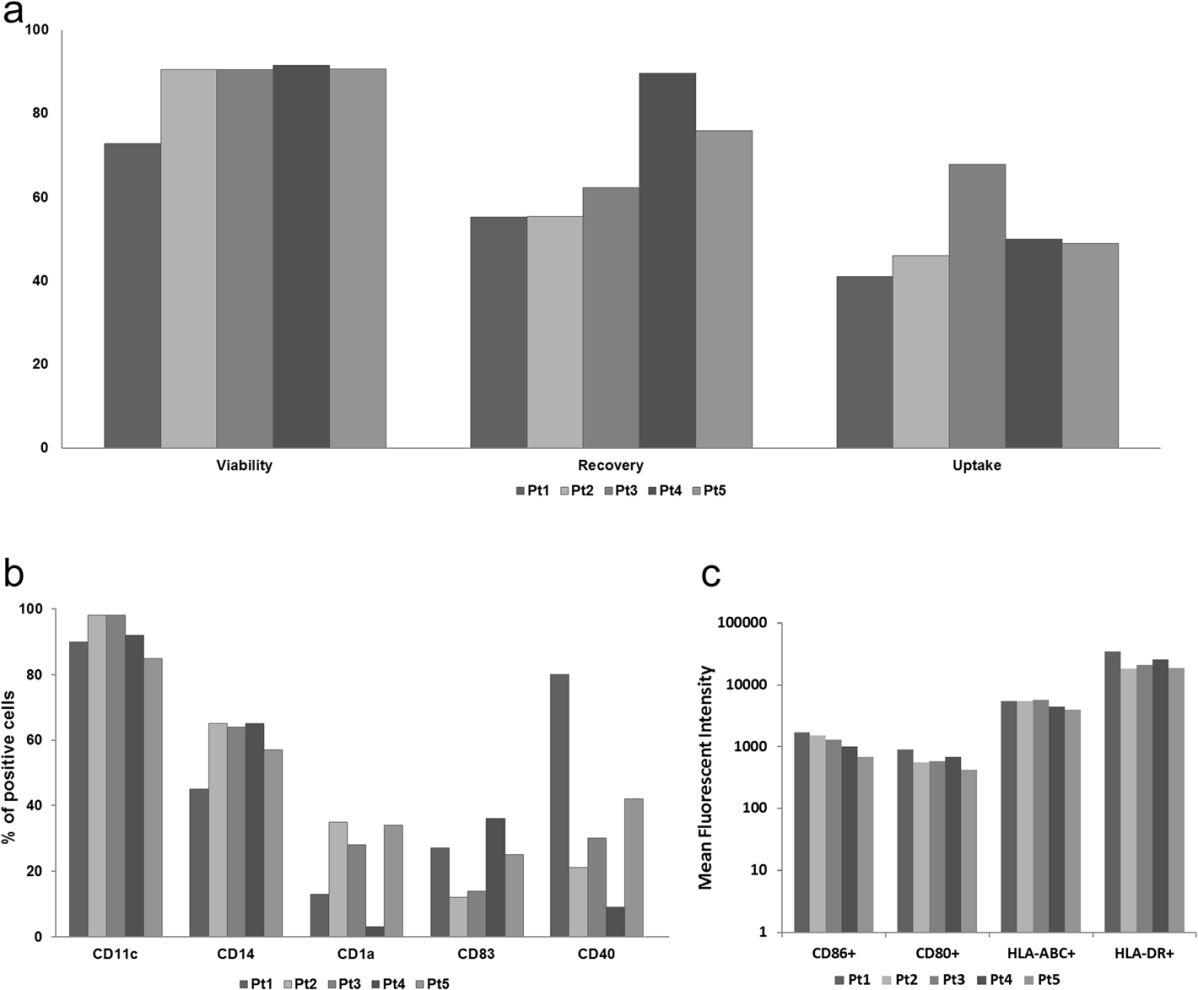
Characterization of the patients’ IFN-DCs used in the clinical trial. IFN-DCs were checked for viability and recovery and capability of antigen uptake**(a)**, and characterized for typical differentiation and activation markers **(b)** as well as class I-II and costimulation molecules **(c)**.

Monocyte-derived IFN-DCs generated from each patient were characterized according to release criteria (see Materials and Methods) for cell viability, cell counts, cell phenotype and antigen uptake on cryopreserved aliquots. As shown in Figure 2a and b, IFN-DCs generated for the 5 patients met all release criteria. In fact, viability ranged between 73% and 91% (release threshold 70%); cell recovery was in the range of 55-90% (release threshold 50%); and phagocytic activity averaged in the range of 40-50% with IFN-DCs of patient 3 showing approximately 70% (release threshold 30%).

Phenotypic analysis showed that IFN-DCs from all patients displayed significant expression of class I and class II molecules, co-stimulatory receptors CD80 /CD86 and CD11c marker. IFN-DCs also retained CD14 expression at the same level among different patients, but showed more variable levels of CD83, CD1a and CD40 (Figure 2b).

**Figure 3:**
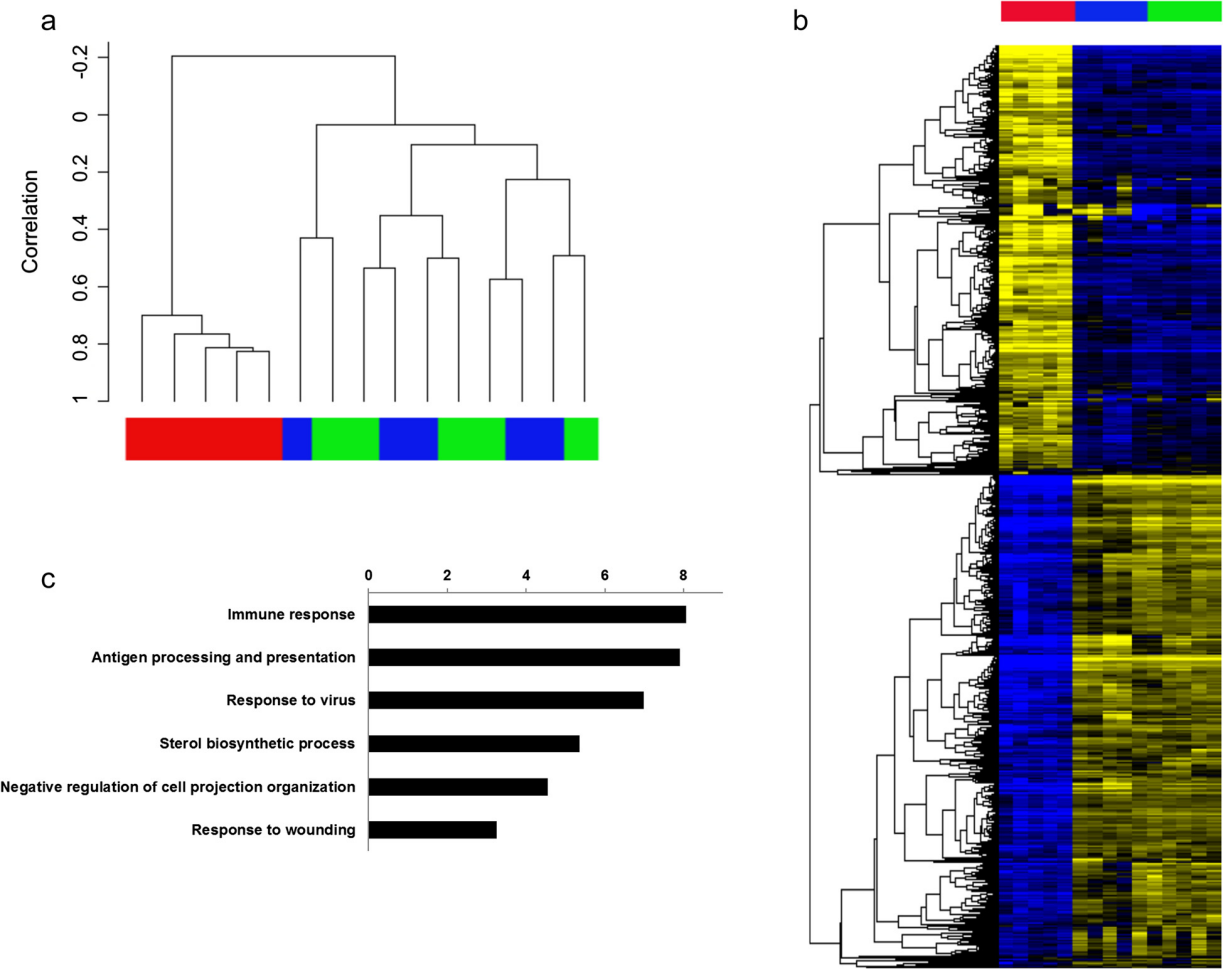
Gene expression analysis of IFN-DCs. **a**. Unsupervised Hierarchical Clustering of samples using the whole dataset. Monocyte, IFN-DCs and prior to cryopreservation DCs are shown by red, green and blue bars, respectively; **b)** Heatmap of the average corrected expression levels of the 5725 genes differentially expressed between IFN-DCs and monocytes with a p-value < 0.001. Genes are in rows and samples in columns. Monocyte, IFN-DCs and prior to cryopreservation DCs are shown by red, green and blue bars, respectively; **c)** Gene Ontology Analysis of up-regulated genes in IFN-DCs vs monocytes (p-value < 0.001 and ratio >3). The plot show for each GO “biological function” term the enrichment among genes up-regulated in IFN-DCs expressed as –log10(p-value). Enrichment p-values were calculated through hypergeometric test. Statistical significance threshold for hypergeometric test was set to 0.05 (i.e., −log10(p-value) > 1.3 were statistically significant).

Then, to more deeply characterize our cell products and monitor molecular changes occurring during the manufacturing process, we also analyzed gene expression profiles of starting monocytes, of DCs at the end of the culture before cryopreservation, and of one aliquot of the thawed DCs product from each of the five patients for which DCs were manufactured. As shown in Figure 3a, unsupervised clustering of the whole dataset clearly separated monocytes from DCs, showing that a huge change occurred during differentiation of monocytes into IFN-DCs. Also, DCs clustered according to patient, implying that changes occurring along cryopreservation and subsequent thawing of the cells are little compared to inter-patient differences and insignificant when considering changes occurring during DC differentiation, which was the main focus of our microarray analysis.

Therefore, to better characterize molecular pathways affected during IFN-DC differentiation, we performed a paired class comparison between monocytes and IFN-DCs and observed that 5,725 genes were differentially expressed with a p-value < 0.001 (false discovery rate < 0.01) (Figure 3b and Table 2). As expected, among top up-regulated genes there were many well-known IFN-α-induced ones, such as ISG15, MX1, IFI27 and IFIT1. Interestingly, several chemokines, such as chemokine (C-C motif) ligand 13 (CCL13), CCL17 and CCL19, were all strongly up-regulated showing fold changes above 100, suggesting a strong chemotactic potential of IFN-DCs towards T cells and other immune cells. Then, to classify genes induced by IFN-DC differentiation, we performed gene ontology (GO) analysis on genes up-regulated in IFN-DCs compared to monocytes, focusing on mostly modulated genes (fold change >3) (Figure 2c). Most over-represented families were immune related with a strong up-regulation of genes belonging to “antigen processing and presentation” and “response to virus”. Considered the relevance of these GO families, we looked at exactly which genes were in our analysis falling into these families. Up-regulated “antigen processing and presentation” genes were mainly class II HLA genes and CD1 genes (a, b, c, and e), highlighting the well-documented ability of IFN-DCs to strongly process and present antigens. Altogether, these data indicated that strong molecular changes are induced upon monocyte differentiation into IFN-DCs and that GMP-manufactured IFN-DCs were empowered, at least at gene expression level, with strong chemotactic and antigen processing and presentation abilities.

**Table 2:** **20 most up-regulated genes in IFN-DCs vs monocytes**

**Symbol**	**Name**	**IFN-DCs /mono expression level**
CCL17	chemokine (C-C motif) ligand 17	10480.71
FPR3	formyl peptide receptor 3	1192.98
LAMP3	lysosomal-associated membrane protein 3	618.05
GGT5	gamma-glutamyltransferase 5	409.08
IFIT1	interferon-induced protein with tetratricopeptide repeats 1	229.97
IFI27	interferon, alpha-inducible protein 27	195.23
CCL19	chemokine (C-C motif) ligand 19	144.92
IFITM1	interferon induced transmembrane protein 1	139.12
MX1	myxovirus (influenza virus) resistance 1, interferon-inducible protein p78 (mouse)	133.25
CCL13	chemokine (C-C motif) ligand 13	123.96
ISG15	ISG15 ubiquitin-like modifier	114.72
IFI6	interferon, alpha-inducible protein 6	105.97
CLEC10A	C-type lectin domain family 10, member A	100.71
LAD1	ladinin 1	99.93
MMP12	matrix metallopeptidase 12 (macrophage elastase)	95.58
GSN	gelsolin	80.37
NUP62	nucleoporin 62 kDa	80.03
TIFAB	TRAF-interacting protein with forkhead-associated domain, family member B	76.55
RNASE1	ribonuclease, RNase A family, 1 (pancreatic)	73.2
RASAL1	RAS protein activator like 1 (GAP1 like)	70.97

### Evalutation of toxicity of the IFN-DCs/DITC regimen and clinical outcome

No severe side effects (Grade III/IV, National Cancer Institute Common Toxicity Criteria version 4) were observed. Grade I toxicity resulted in pain in the site of DC injection, lasting less than 30 min. After the completion of the programmed therapy, patients 1 and 3 developed signs of autoimmunity (vitiligo). Clinical response was not a primary end point of the current study. Although no major responses were observed, all three patients experienced disease stabilization (7, 26 and 5 months for patients 1, 3, and 5, respectively). After progression, patient 1 underwent amputation of the anterior part of the foot where melanoma lesion progressed and she is still alive (OS 34 months). Patient 3 progressed after 26 months and is currently alive and being treated with Pembrolizumab 2 mg/Kg every 3 weeks, into an expanded access program (OS 31 months).

### Characterization of the immune response

**Figure 4:**
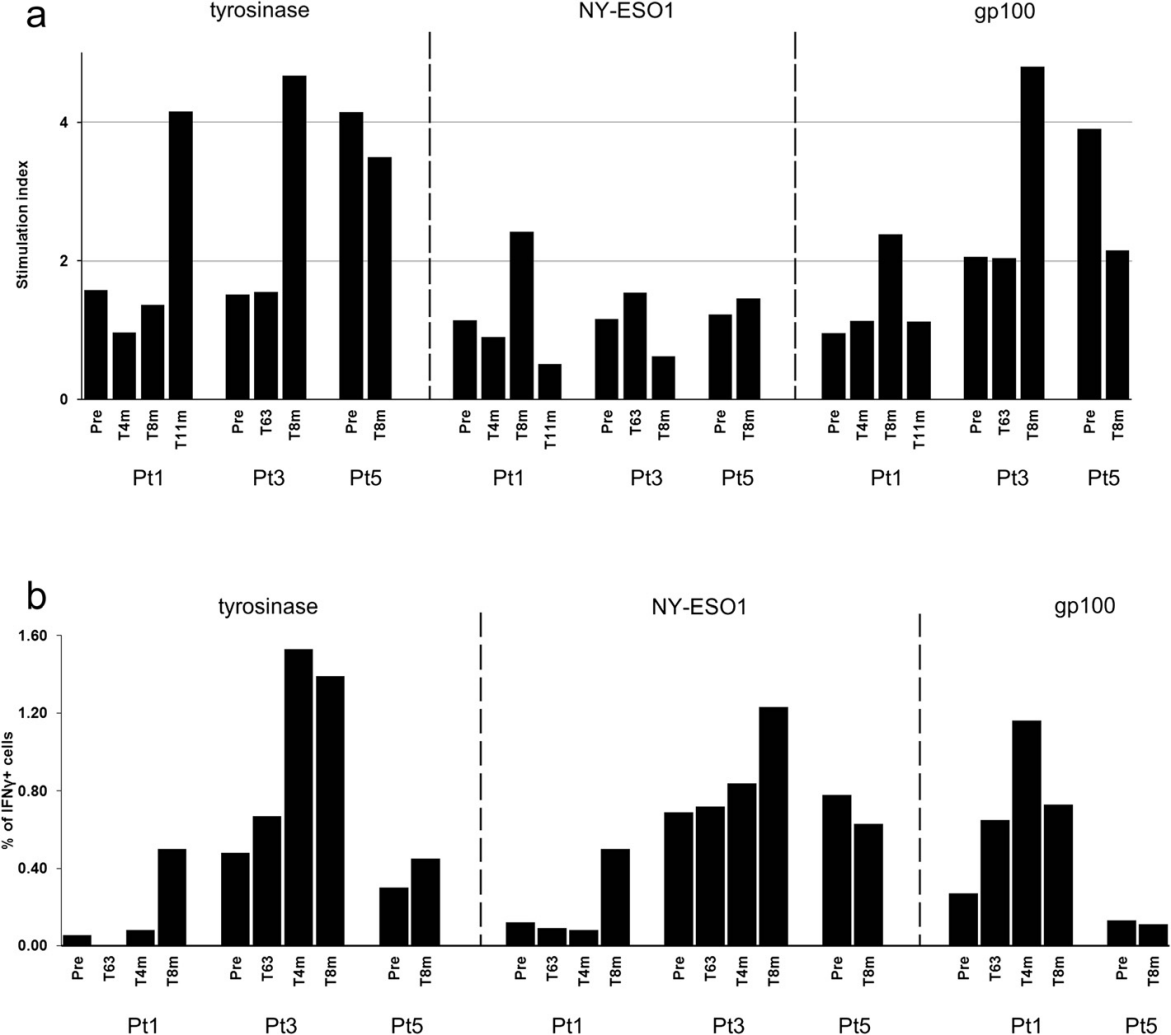
Characterization of immune responses of patients that completed the treatment. **a**. Lymphoproliferation assay of PBMCs collected at indicated time points and stimulated with different melanoma-associated antigens (NY-ESO-1, tyrosinase, gp100, MART-1, survivin, MAGE-A1, and MAGE-A3). Proliferative activity was reported only for antigen-positive culture. Pre: prevaccination time; m: months. **b**. Intracellular cytokine staining (ICS) performed on T cell lines derived from *in vitro* expansion of PBMCs collected at different time points during treatment, with the tyrosinase, NY-ESO-1 and gp100 peptide pools. Histograms represent percentages of IFN-γ positive cells assessed within the CD3 + CD8+ gate after 6 h stimulation with IFN-DCs pulsed with the indicated peptides pools. Unpulsed IFN-DC were used as a control at each time point and for each antigen resulting in almost undetectable or very low levels of IFN-γ positive cells (% ranging from 0.02 to 0.08) for all samples, except in the case of both NY-ESO-1-specific samples (0.2%) from Pt5. Cells were analyzed by flow cytometry using a FACS DIVA and FlowJo software (version 10; TreeStar).

The immunological monitoring was focused on assessing whether the administration of IFN-DCs could result in activation of tumor-specific T cells. Considering that the DCs used were injected intratumorally with the aim of *in situ* taking up antigen of DTIC-induced apoptotic tumor cells, we decided to test reactivity of T cells against a broad range of well-known melanoma associated antigens (i.e., MART-1, NY-ESO-1, MAGE-A1, MAGE-A3, gp100, tyrosinase and survivin). Thus, we set up a lymphoproliferation assay by culturing patient’s PBMCs, collected before and at different times after treatment, in the presence of overlapping peptide pools, each related to a specific melanoma-associated antigen and spanning the whole protein sequence. We carried out the analysis on the 3 patients that completed the planned DC-based regimen. As depicted in Figure 4a, patient 1 showed lymphoproliferative response to tyrosinase, gp100 and NY-ESO-1 usually occurring at times after treatment (i.e., 8 or 11 months). Patient 3 showed a response to gp100 already on prevaccination sample, but did exhibit the induction of a lymphoproliferative response against tyrosinase and an increase against gp100 at 8 months. Responses against tyrosinase, gp100 and MAGE-A1 (not shown) were already present in Patient 5 in prevaccination samples and remained unaltered for tyrosinase, while it was markedly reduced for gp100 after treatment. We were unable to detect any response before and post-treatment towards MART-1, MAGE-A3 and survivin (data not shown).

In order to expand the response to these tumor antigens, we performed an *in vitro* sensitization of patient’s PBMCs with those peptide pools showing response in the lymphoproliferation assay. Thus, we analyzed specificity, frequency and effector function of T cells after *in vitro* sensitization. As shown in Figure 4b, cells from both patient 1 and 3, although at different levels, showed an increase in the percentage of CD8+ cells producing IFN-γ in response to both tyrosinase and NY-ESO-1 compared to prevaccination levels , while cells from patient 5 did not show any increase to these antigens. Similar conclusions can be traced on levels of CD69 expression (data not shown). Of note, a strong production of IFN-γ in response to NY-ESO-1 (especially in CD3^+^CD4^+^-gated cells) was already present in patient 5 before treatment with IFN-DCs and was maintained at 8 months. Further analysis performed by multimer staining specific for some tumor antigens, revealed that patient 1 showed no increase of multimer^+^ cells overtime (data not shown). In patient 3, the proportions of CD8^+^Tyrosinase^+^ e CD8^+^MART-1^+^ cells were transiently expanded. In patient 5, the analysis indicated an higher percentage of NY-ESO-1 specific tetramer^+^ cells over time (data not shown).

**Figure 5:**
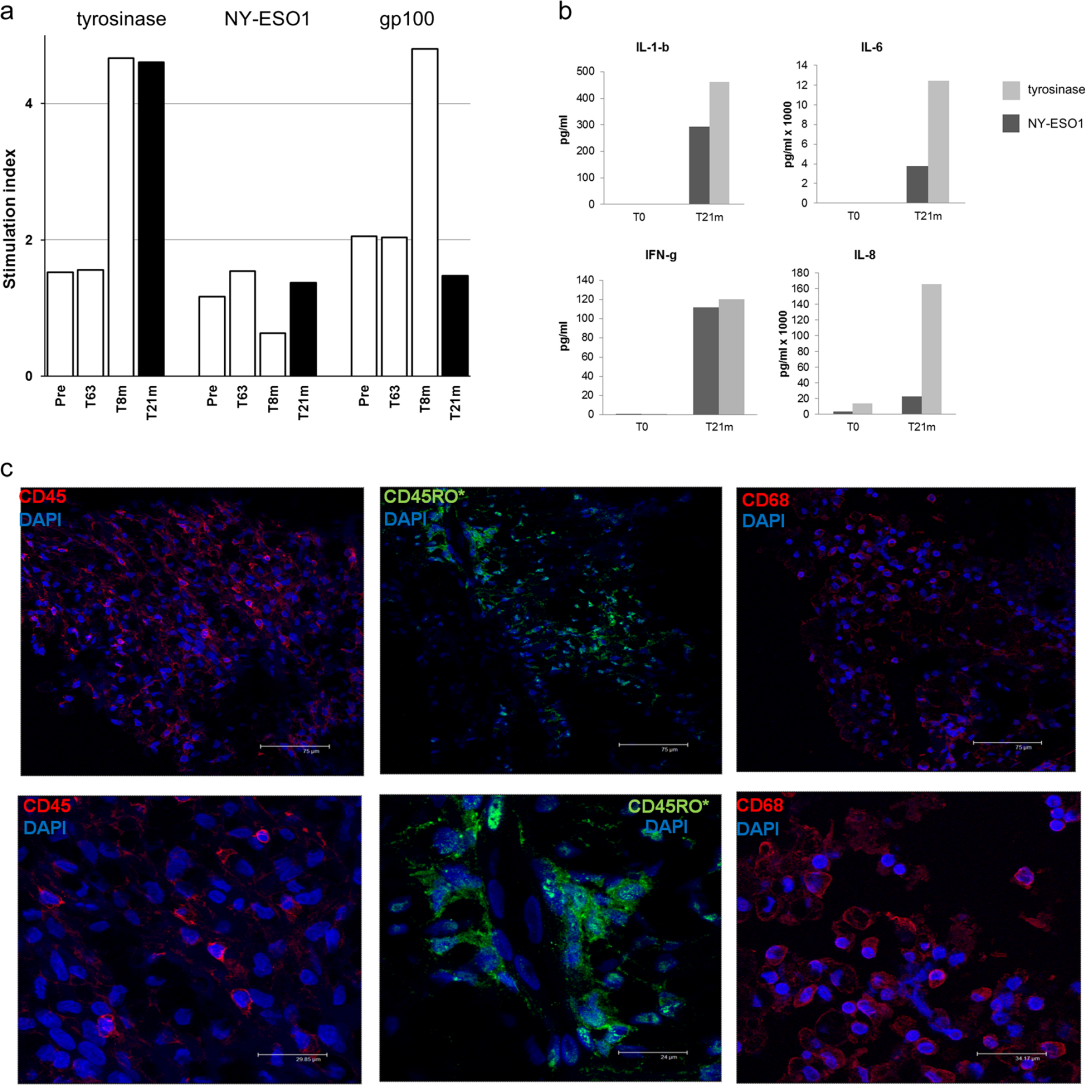
Long term immune responses of patient 3 showing durable disease stabilitazion. **a**. Lymphoproliferation assay in response to antigen stimulation of PBMCs from pt3 collected at T21m, compared with results obtained from the previous tests. **b**. Lymphocyte cultures (the same as in panel **a)** analyzed for cytokine production by Bio-Plex® Multiplex System. Only cytokine-positive antigen-specific cultures are shown. Negative controls showed no detectable cytokine levels except for IL-8-specific samples (2.6 × 10^3^ pg/ml and 3 × 10^3^ pg/ml for, respectively, time points T0 and T21). **c**. Immunofluorescence of biopsy from pt3 metastatic lesion. The tumor sample was collected 1 month after the last evaluation of patient clinical status by PET (T21m). The immunofluorescence microscopy was done using antibodies specific for CD45, CD45RO, and CD68 stained with 4′,6-diamidino-2-phenylindole (DAPI). Images show that tumor lesion is abundantly infiltrated both by T lymphocytes **(a)** which are characterized by a memory phenotype **(b)** and a monocyte/macrophage population **(c)**.

It is worth noting that, among the treated patients, patient 3 showed an overall good immune response to some relevant tumor peptides (Figure 4a and b) as well as a durable disease stabilization (Table 1). Approximately 21 months after enrolment, patient 3 underwent disease evaluation by PET/CT scanning, confirming the status of disease stabilization. Therefore, we took the chance to analyze at this time both the immune status and the lymph node biopsies. As illustrated in Figure 5a, PBMC from patient 3 retained at this time point a lymphoproliferative response to tyrosinase, while the response to gp100 was reduced to pre-vaccination levels and the one to NY-ESO-1 remained stable. We also assessed T cell activity and effector function by measuring production of several cytokines in the same cultures used for the lymphoproliferation assay. As shown in Figure 5b, we found appreciable amounts of the inflammatory cytokines IFN-γ, IL-1β, IL-6 and IL-8 in cultures stimulated by tyrosinase and NY-ESO-1, while no cytokines were detectable in other peptide-specific cultures (data not shown). Levels of other cytokines (i.e., IL-2, IL-4, IL-10, IL-12, IL-17A, TNF-α and GM-CSF) did not reach minimum detection threshold in any of the antigen-specific cultures. In addition to this, we analyzed immune cell infiltrates in tumor tissues collected at this point from patient 3. As shown in Figure 5c, abundant tumor infiltrating lymphocytes were present (left panel), which were characterized by memory phenotype (central panel). Also, a large number of monocytes/macrophages was present (right panel).

## Discussion

Here, we report the results of a phase I clinical study evaluating a new chemo-immunotherapy approach based on intratumoral injection of unloaded IFN-DCs one day after DTIC in patients with advanced melanoma. The rationale was to combine tumor cell death induced by the chemotherapeutic agent with the phagocytic activity and T-cell stimulatory properties of IFN-DCs, therefore aiming at an *in vivo* loading of DCs with patient specific tumor associated antigens. The chemo-immunotherapy was well tolerated and the induction of antigen specific immune responses was observed in two out of the three patients that completed the protocol treatment. In particular, one patient was revaluated at 21 months and antigen specific immune response was still detectable and proved to be associated with high infiltration of immune cells inside the tumor lesion.

Intratumoral injection of DCs combined with tumor cell death inducing agents or therapies has already been tested and proved effective in animal models [32,36,37,47-49]. Also, clinical studies have been performed combining DCs with radiotherapy or monoclonal antibody in some clinical settings showing safety and feasibility of the approach [33,38,39,50,51]. Our study is the first showing safety and feasibility of IFN-DCs injected intratumorally one day after DTIC. IFN-DCs have been widely characterized *in vitro* and in animal models for their peculiar phenotype and functional properties [12,14-16]. In this study, we characterized the gene expression profiling of monocytes and IFN-DC from patients, showing that during DC differentiation/maturation process IFN affects significantly mainly genes involved in antigen processing and presentation as well as activation of immune responses, in agreement with previous findings [12,14]. Our results also confirmed the partially mature phenotype of these cells as well as their strong phagocytic and chemotactic activity. All these features may have played a key role in our setting by overcoming tumor microenvironment immunosuppressive signals [52-54] while maintaining the necessary high phagocytic activity instrumental for processing antigens released by apoptotic tumor cells. In line with all the published results from clinical studies based on standard IL-4-conditioned DCs, also IFN-DCs were well tolerated and only grade I toxicity was observed in treated patients. On the other hand, also DTIC may have played a key role in our setting. In fact, recent studies have shown its immunogenic properties, mainly triggering or enhancing the expression of NKG2D ligands on tumor cells, thus promoting NK-cell cytotoxicity and IFN-γ-dependent tumor specific T cell activation [55]. These observations, together with the evidences of clinical and immunological response observed in the three treated patients warrant future clinical studies to evaluate efficacy of this chemo-immunotherapy and better characterize its mechanism of action.

The analysis of immunological response of patients to tumor antigens is critical for correct evaluation of the efficacy of chemo-immunotherapeutic approach. However, differently from pulsed-DC-based settings, where testing reactivity against pulsed antigens is relatively easy, in the setting of *in situ* loading the antigens processed and presented by DCs are unknown. Here, by testing a broad range of well-known melanoma associated antigens, we had evidences of immunological activation against tyrosinase, gp100 and NY-ESO-1 in the two patients showing long-term stable disease. It is also noteworthy to mention that both these patients also experienced vitiligo. Vitiligo is an autoimmune condition due to immune-dependent destruction of melanocytes; and development of vitiligo in melanoma treated patients has a good prognostic value [56,57]. The exact mechanisms of melanoma-associated vitiligo development are still under study, but it is clear that CD8^+^ T cell recognizing autoantigens have a major role [58] and that tyrosinase and gp100 are among these autoantigens [59]. Strength and relevance of this association (i.e., induction of T cell response against tyrosinase and gp100 and development of vitiligo) in our setting will be evaluated more in details in future studies with larger cohorts of patients.

Evaluation of clinical efficacy was not among the study endpoints. However, we did observe long-term disease stabilization in patient 3. The patient was enrolled after progressing from three different therapeutic approach: standard DTIC, Vemurafenib and Ipilimumab. At time of this writing, the patient is still alive at 31 months from the enrollment into this study; in particular, after 26 months of disease stabilization, due to disease progression he started therapy with Pembrolizumab as part of an expanded access program. Whether previous therapies had a role in this response is impossible to evaluate. Kinetics of response with Ipilimumab after initial progression have been described, usually occurring few months after treatment [60,61]. The patient was treated with ipilimumab 6 months before enrolment and showed disease progression (lymphonodal and lung) by modified WHO criteria after 26 months [62].

## Conclusion

In conclusion, here we report the results of a phase I clinical study testing a chemo-immunotherapeutic approach based on IFN-DCs injected intratumorally one day after DTIC in advanced melanoma. The treatment was well tolerated, and clinical and immunological responses, including development of vitiligo, were observed, therefore warranting additional clinical studies aimed at evaluating efficacy of this approach. Other than hold promises for this setting, this approach should also be evaluated for other solid tumors by selecting strong immunogenic cell death agents to be combined with patient-derived IFN-DCs.

## Abbreviation

DTIC: Dacarbazine
DCs: Dendritic Cells
IFN: Interferon
NK: Natural Killer
Th-1: T-helper-1
TLRs: Toll-like Receptors
